# Participation in community seniors' organizations and mental health among retired adults in urban China: The mediating role of interpersonal needs

**DOI:** 10.3389/fpubh.2022.1045948

**Published:** 2022-12-21

**Authors:** Yuruo Lei, Jie Lao, Jiawei Liu

**Affiliations:** Global Megacity Governance Institute, Shenzhen University, Shenzhen, China

**Keywords:** participation in community seniors' organizations, mental health, positive emotions, negative emotions, social capital, interpersonal needs, retired adults, urban China

## Abstract

**Purpose:**

This study aimed to investigate the association between participation in community seniors' organizations (CSOs) and mental health among retired adults in urban China, and illustrate the causal mechanism.

**Methods:**

We collected data on participation from a community seniors' organization and mental health survey in Shenzhen City, China, in July 2022. The survey used a two-stage cluster sample design, based on administrative divisions as the primary sampling unit and communities as the second sampling unit, where retired adults were randomly sampled. The inclusion criteria were individuals >60 years old (the mandatory age for retirement in China) or women who retired early at the age of 45 years. We used three multivariable regression models to estimate the effects of participation in CSOs on mental health. Furthermore, we used structural equation models to examine the mediator of acquired interpersonal needs in the association between CSOs participation and mental health.

**Results:**

The study examined the values of CSOs, generated explicitly for older adults and explained how participation in such organizations benefits retired adults' mental health. Mental health is defined in three dimensions: aggregate mental health, positive emotions, and negative emotions. The results show that constituting social networks with like-minded individuals and perceiving interpersonal needs are the two main benefits of CSOs on mental health. The retired individual who participated in CSOs with a higher level of diversity and frequency, joined specific types such as health- and study-related CSOs, could receive more interpersonal needs and were probably mentally healthier. The mediating mechanism of interpersonal needs associated with participation in mental health was significant. Besides, mental health is generally affected by physical health, sleep quality, and socioeconomic status.

**Conclusion:**

This study suggested that CSOs have expanded the social interaction channels of retired adults and affected their mental health by providing basic interpersonal needs such as inclusion, dominance, and affection. Among the types of CSOs, health and study organizations might enhance mental health most effectively, while semi-official organizations have no effect.

## Introduction

Mental health issues or distress (e.g., depression, anxiety, and poor sleep quality) (1–6) have been increasingly recognized as one of the most popular public health problems worldwide across different age groups including aging population (7–12). In particular, such mental health problems among older adults have placed a huge burden on society, public health, and the medical system (13–16). Previous studies have indicated that isolation, loneliness, and/or mental distress often occur with a decline in physical and cognitive function in late life (17–20). The authoritative data has indicated that more than 20% of the world population aged 60 years and above suffer from mental disorders (21–23). Among retired adults with chronic disease, 35–40% have mental health comorbidity (24). As the largest segment of the world's retired adult population, Chinese retired adults aged 65 years and above exceeded 164.49 million in 2019 (25). Retirement may increase depressive symptoms in adults, especially in men from lower occupational class backgrounds (26). A previous study has shown that retirement could lead to the 50–75 aged adults being overwhelmed by self-perception of retirement (27). The rate of depression among retired adults is 6–29.4%, and those with physical diseases is 52.1–63.4% in China (28). Thus, mental health in retired adults requires more attention.

Promoting social participation is one of the key proposals of “active aging (29) and other age groups like adolescents (3, 6, 30, 31) and the Chinese government has adopted this policy as one of the important means of achieving successful aging for older adults in 2019. Numerous studies have shown that social participation influences mental health (32–34). Social participation could broaden social interactions and reduce psychological problems caused by changes in life, physical conditions, and social relationships (35). This could reduce the risk of depression (36) and greatly improve mental health. Furthermore, the effect of social participation on mental health in retired adults was significantly higher than that in younger adults. However, most studies have examined the effect of participation in social and leisure activities (37). Few have examined the effect of community seniors' organizations (CSOs) participation. Furthermore, the mechanism linking CSOs participation and mental health remains understudied.

Retired adults often report feelings of social isolation, loneliness, anxiety, or depression because their social roles and ties in social connection networks have significantly changed. Furthermore, a deficient sense of belonging to the new social role and social context leads to negative effects such as a loss of self-fulfillment due to not working and socializing with colleagues (38). China has a collectivist culture, and retired adults often meet to share daily activities in local communities (39–41). Emerging evidence suggests that mental health can be enhanced through participation in CSOs (42). These organizations are common government-financed community social organizations in urban China like Shenzhen City, and may be important in linking social interaction with mental health, considering the high needs of retired adults for social belonging and interpersonal needs. In this study, we applied a social capital theory approach to explore whether CSOs financed by the government can improve retired adults' mental health and the underlying mechanisms among retired adults in urban China.

## Literature review

### Social capital theory

Social capital theory forms the theoretical bedrock for analyzing the effect of CSOs participation on the mental health of retired adults. Social capital first emerged from research by Durkheim in 1980 on the importance of building strong families and local communities. According to Durkheim, social capital is a social relationship formed by an institutionalized network of mutual acquaintances and recognition, and a collection of actual or potential resources that provide benefits and convenience to members (43). Putnam (44) defined social capital as “features of social organizations, such as networks, norms, and trust, which facilitate coordination and cooperation for mutual benefit”.

According to Durkheim and Putnam's definitions, social capital has two fundamental features. First, the sources of social capital stem from the structure of social organizations rather than attributes of individuals. Second, social organizations are places where individuals acquire social networks through participation. Generally, a society with high levels of social capital is characterized by high levels of social interaction, trust in others, and reciprocity, resulting in enhanced interactions with others (45).

A CSO is described as a community-level organization that is partly financed by the government, but operated by a non-governmental organization and recognized as social capital in China. First, it was established to pursue a specific purpose: to meet the diverse demands of retired adults, in which retired adults are the main participants. Second, it has the characteristics of a social organization, such as low transaction costs, high organizational recognition, and mutual trust among organizational members (46). These characteristics enable CSOs to have a specific social relationship network, which helps promote the formation of retired adults' participation intention. Third, compared to other community social organizations, CSOs display homogeneity, alignment, and interests of retired adults and can effectively meet retired adults' diverse needs in China's urban community.

### Participation in CSOs and mental health

Participation in CSOs can be described as an individual's involvement in organizations for older adults that form social networks and provide opportunities for social interaction among retired adults in a community.

Participation is an important mechanism by which a community-level organization affects the mental health of retire adults. Studies have shown the benefits of social interaction in community-level organizations on mental health. For example, a longitudinal study in Indonesia found that the number of community-level organizations was positively associated with self-reported physical and mental health (47). Some studies have found that the type of participation in community-level organizations (volunteer activities, community events, clubs for the elderly) could bring mental health benefits to retired women irrespective of an individual level relationship (e.g., friendship, communication with family and friends, hobbies) (48). Croezen et al. (34) found that participation in religious organizations offered mental health benefits beyond those offered by participation in other forms of social organization. Broad social interaction and regular collective leisure activities might have beneficial effects on the functional health of retired adults through behavioral and mental pathways (49).

Building on the above studies, we may assume that CSOs have expanded social networks with like-minded people and broadened the social interaction channels of retired individuals in China. This bridges and enhances social inclusion, to enable positive effects on the mental health of retired adults. Furthermore, the diversity, frequency, and quality of participation in CSOs, and types of CSOs help these individuals to understand their mental health.

### The mediator role of interpersonal needs in the association between participation in CSOs and mental health

Interpersonal needs refer to the desire for affection/openness, control, and inclusion when individuals get together in a group, which is theorized from social relationships (50). It is common knowledge that humans need to form close affectional ties (51), and social interaction and interpersonal connections provide individuals with a sense of belonging and social identity, and opportunities to participate in activities and projects (52). However, retired adults tend to experience a decline in physical and cognitive function as they age. Social networks may shrink, which may lead to social isolation. Social isolation and inactivity increase the risk of a first episode of major depressive disorder (53). Additionally, retired adults who are socially isolated have a much greater risk of dying than those who are well connected (54). In contrast, retired adults who possess multiple social roles can boost their self-esteem and self-worth (55), which meets their interpersonal needs to some extent. For example, one study indicated that retired adults who participated in volunteer activities perceived themselves to be in good self-rated health and had fewer depressive symptoms and good functional abilities, possibly because volunteering provides them with the opportunity to adopt new social roles that are mentally rewarding (56). These findings indicated that meeting interpersonal needs may be beneficial to the mental health of retired adults. In summary, CSOs provide social interaction and social role-acting opportunities and enhance retired adults' social sense of belonging. Thus, they may promote their mental health by meeting interpersonal needs.

Although previous studies have examined the effect of participation in community-level organizations and interpersonal needs on retired adults' mental health, some research gaps remain. First, existing studies are mainly based on the effect of social interaction. Few studies have directly examined the association between participation in community-level organizations and retired adults' mental health, and underlying mechanisms have not been fully examined. Furthermore, no study has examined the mediation mechanism among participation in community-level organizations, interpersonal needs, and mental health in China. Based on survey data, this study examined the mediating role of interpersonal needs in the relationship between CSOs participation and mental health among retired adults in urban China, which provides an approach reference for China's active aging.

Building on these studies, CSOs provided places of participation and interaction, and offered a sense of belonging where participants could make friends and engage in various activities, which reduces their risk of being gradually left behind by society. Therefore, we hypothesize:

H1: CSO participation has a significant effect on the mental health of retired Chinese adults.

H2: Interpersonal needs mediate the relationship between CSO participation and mental health among retired adults in urban China.

## Methods

### Participants and sample

The data were sampled using a two-stage cluster sample design and collected directly in Shenzhen, China, in July 2022. In the first stage, five communities were sampled randomly. In the second stage, cluster sampling was used to survey the members and participants of the community seniors university (laoniandaxue)^1^ and community seniors care centers (laonianren zhaoliao zhongxin) where trained interviewers conducted interviews. Under the Chinese top-down policy of promoting purchase of service contracting (POSC) from non-profit organizations, community care centers have gradually become the most important activities for retired individuals to exercise, entertain, make friends, and volunteer services. Therefore, community care centers were used as the final sampling unit to obtain a representative sample. In total, 193 retired adults were surveyed using cluster sampling. Of these, five returned incomplete questionnaires with several missing responses and 188 (97.4%) responded. The participants were 44–76 years old (70.7% women), with a mean age of 62.61 years and SD of 6.28.

### Inclusion and exclusion criteria

The inclusion criteria were >60 years or women who retired early at the age of 45 years. The exclusion criteria were < 60 years but not retired, inability to understand or complete the questionnaire because of serious disabilities, and lack of informed consent.

### Measures

#### Mental health

We used 5-items from the China Health and Retirement Longitudinal Study Wave 4 (2018) Questionnaire to measure mental health (e.g., “No matter what happens I can adjust my status”; “Feel as happy as when I was young”) (57), as shown in Table 1. Responses were based on a 5-point rating scale (1 = “never” to “5 = always).” The scores of the last three questions were reversed, with higher scores indicating higher mental health levels. In this study, Cronbach's alpha for the scale was 0.612.

**Table 1:** Mental health scale.

	**No matter what**	**Feel as happy**	**Often feel**	**Often feel**	**I often feel that the**
	**happens, I can adjust**	**as when I was**	**afraid**	**lonely**	**older I get, the less**
	**my status**	**young**			**useful I am**
Never (%)	21.74	4.79	52.13	56.38	44.15
Sometimes (%)	18.48	14.89	34.57	30.32	28.72
Often (%)	11.41	20.21	9.57	10.64	15.43
Usually (%)	30.43	38.83	2.66	1.60	8.51
Always (%)	17.93	21.28	1.06	1.06	3.19
Average (1–5)	3.0	3.6	4.3	4.4	4.0

We used principal component analysis to process 5-items of mental health to acquire a better understanding of the potential internal structure of retired adults' mental health (58). The equation is transformed factor values = (factor + B) × A, A = 99/(factor maximum–factor minimum), and B = (1/A)–factor minimum. After rotating by the maximum variance orthogonal method, two common factors “positive emotions” and “negative emotions” were identified. Thereafter, we converted the standardized factor scores of these two factors into an index ranging from 1 to 100. The second and third independent variables were “Positive emotions” and the third “Negative emotions.” As shown in Table 2, the aggregate mental health of the retired sample was 19.4, with a minimum value of 8 and a maximum value of 25. After the “1–100” conversion, the positive and negative emotions were 55.6 and 22.7, respectively.

**Table 2:** Description of variables.

**Variable code**	**Variable content**	**Obs**	**Mean**	**Std. dev**.	**Min**	**Max**
MH	Mental health	188	19.404	3.227	8	25
PE	Positive emotions	188	55.6	22.9	1	100
NE	Negative emotions	188	22.7	16.4	1	100
Gender	Gender (man = 1, women = 2)	188	1.707	0.456	1	2
lnage	Logarithm of age	187	4.131	0.102	3.784	4.331
Edu3_1	Primary school or below	188	0.176	0.381	0	1
Edu3_2	Junior high school, high school, technical secondary school	188	0.489	0.501	0	1
Edu3_3	College degree or above	188	0.335	0.473	0	1
Physical health	Subjective physical health	188	2.293	0.973	1	5
Sleep quality	Sleep quality	186	2.672	0.95	1	5
Material wealth	Material wealth	188	2.25	1.805	0	7
SES	Socioeconomic status	182	4.918	1.997	1	10
Diversity	Diversity of participation	188	2.367	1.547	0	7
Frequency	Frequency	180	2.533	1.15	1	5
Quality	Interpersonal needs	188	17.309	5.704	0	25
Hobby-related CSOs	Hobby-related CSOs	188	0.910	0.729	0	1
Health-related CSOs	Health-related CSOs	188	0.431	0.497	0	1
Study-related CSOs	Study-related CSOs	188	0.463	0.500	0	1
Semi-official CSOs	Semi-official CSOs	188	0.484	0.690	0	1

#### Participation in CSOs

Participation in CSOs was measured in four dimensions. **The diversity** was assessed by enquiring whether respondents participated in the following six types of groups in their neighborhood: literature and art, sports, healthcare, comprehensive, learning, and public welfare. Responses to each item were binarized (i.e., respondents who participated in the group were coded as 1; if not, they were coded as 0 for each item), and the mean was 2.4. **The frequency** was accessed by enquiring “how many times do you participate in social organization activities per week on average?” The response options were: < 1 time, 1–2 times, 3–5 times, 6–7 times, and > 8 times, with assign values of one, two, three, four and five, respectively and the mean was 2.5. **The quality** refers to the theory of interpersonal needs and poses five questions from the three dimensions of belonging needs, domination needs, and emotional needs. The five questions were: (1) “I am a member of a social organization; I belong to this group.” (2) “Other members of the social organization can understand me, I agree with me.” (3) “I can convince other members to do what I want.” (4) “I can maintain a very close personal relationship with other members.” (5) “I feel honored when I do something with other members, a great sense of achievement.” The responses were never, rarely, sometimes, often, and always, and they were assigned values from 1–5, and thereafter summed up, reflecting the interpersonal needs from participating in CSOs, with an average value of 17.3 and a maximum of 25. **The types** were assessed by classifying the above six types of social organizations into four categories: Hobby-related CSOs, Health-related CSOs, Study-related CSOs, and Semi-official CSOs. Responses to each item were binarized (i.e., respondents who participated in the group were coded as 1; if not, coded as 0 for each item).

#### Control variables

Based on previous studies' results, this study added the variables: physical health, sleep quality, material wealth, and socioeconomic status as control variables in the regression models, and demographic variables, such as respondent gender, age (natural logarithm), and education level. The descriptive statistical structures of all independent variables were shown in Table 2. The participants were 44–76 years old (70.7% women), with a mean age of 62.61 years and SD of 6.28. We used a single item by Vingilis and Wade to measure **physical health** (59). We used a 5-point Likert scale (1 = very good, 5 = very bad) to measure one item of self-rated physical health status (e.g., “In my opinion, my general health status is…”). The item was reversed on the scale, and total scores ranged from 1 to 5, with higher scores indicating a higher level of physical health. Self-related health is a subjective assessment of health status that has been widely adopted in large-scale surveys, as a well-established predictor of mortality (60). We measured **sleep quality** using a single item by Hicks et al. (61). We used a 5-point Likert scale (1 = very satisfactory, 5 = deeply discontent) to measure one item of subjective sleep quality (e.g., “In my opinion, my general sleep status is…”). The item was reversed on the scale, and total scores ranged from 1–5, with higher scores indicating a higher level of sleep quality. We used six items from the China Health and Retirement Longitudinal Study Wave Questionnaire (57) to measure **material wealth** (e.g., have extra income beyond retirement salary,” “have a car”). The average score was 2.3. We used a family social class rating scale to measure **socioeconomic status** and the mean value was 4.9.

## Results

### Effects of control variables on mental health

As shown in Table 3, we conducted a bivariate correlation analysis to show the relationship between mental health and a set of control variables, including gender, education, material wealth, and socioeconomic status (SES), which were assumed to be causes of variation. Pearson's correlation coefficient, symbolized by a lower case, denotes the direction and strength of the relationship between variables.

**Table 3:** Correlation among mental health-related variables and control variables.

	**Gender**	**Education**	**Material wealth**	**SES**
Mental health	0.201^**^	0.068	0.194^**^	0.410^***^
Positive emotions	0.048	0.062	0.149^**^	0.152^**^
Negative emotions	−0.222^**^	−0.014	−0.126	−0.403^***^

** *p* < 0.05,

*** *p* < 0.01. SES, socio-economic status. The values in the cells are the Pearson's correlation coefficients among variables.

#### Gender

Gender significantly and moderately correlated with mental health and negative emotion. Compared to men, women had significantly higher levels of mental health (*r* = 0.201, *p* < 0.05) and fewer negative emotions (*r* = −0.222, *p* < 0.05). There was no significant difference in positive emotions between men and women.

#### Age

**Figure 1:**
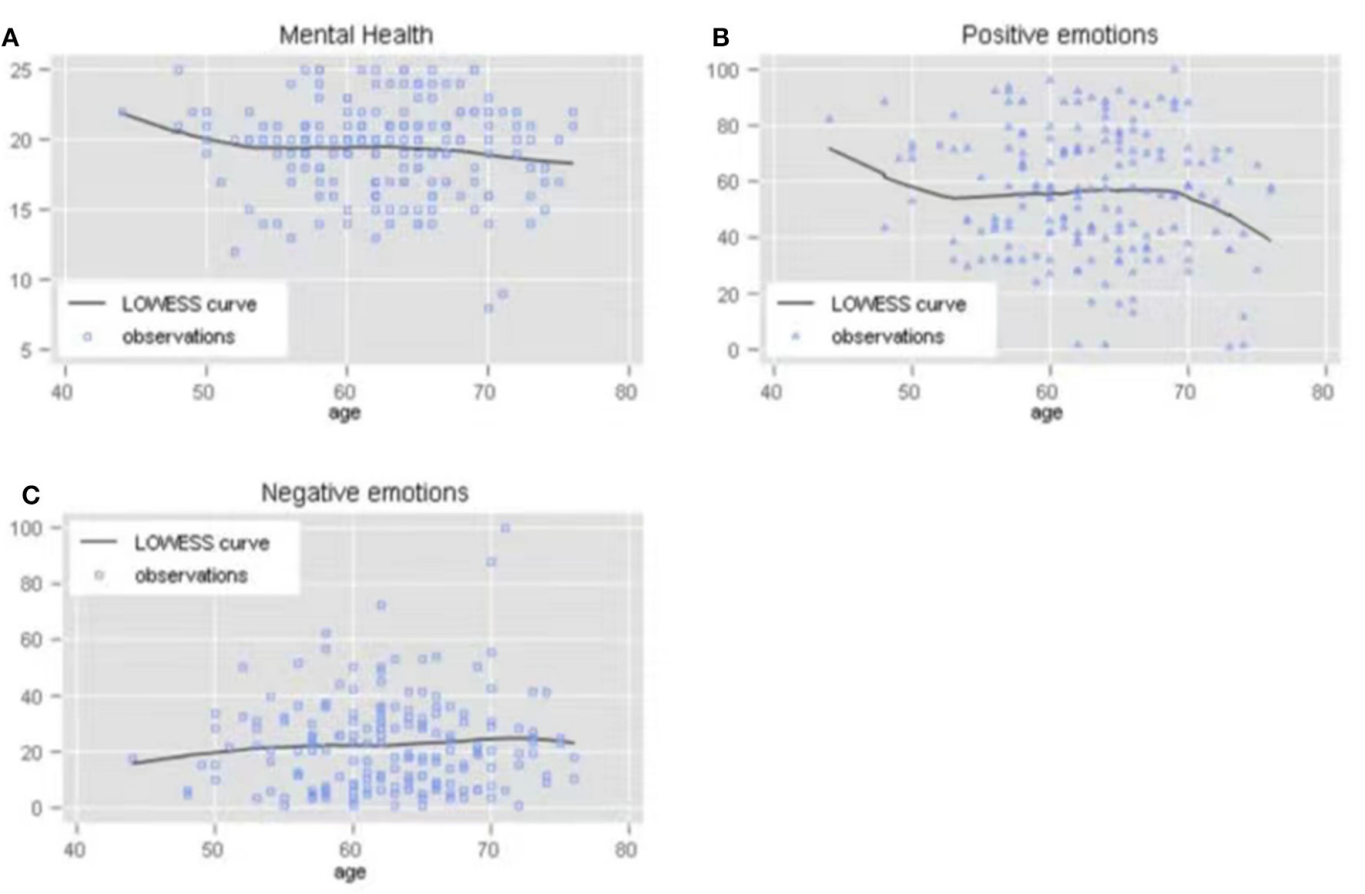
**(A)** The effect of age on mental health. **(B)** The effect of age on positive emotions. **(C)** The effect of age on negative emotions.

As shown in Figure 1, we conducted LOWESS regression analysis to illustrate the association between mental health and age measured in years, denoting a weak non-linear relationship. Compared to respondents < 50 years and >70 years old, those aged between 50 and 70 years were more mentally healthy and positively emotional. When combined with the multivariable regression models, age measured in natural logarithm values, was not significant in any of the models.

#### Education

As shown in Table 3, there was no significant relationship between education level and mental health. The educational levels of retired adults were categorized as “Primary school or below,” “Junior high school, high school, technical secondary school,” and “College degree or above.” There were no significant differences between participants who graduated from primary school and below and those from higher education levels. Education level was not statistically significant in any of the regression models.

#### Material wealth and socio-economic status

Material wealth describes the objective ability to obtain material conditions, while socioeconomic status shows the subjective perception of social class. As shown in Table 3, material wealth significantly and positively correlated with mental health (*r* = 0.194, *p* < 0.05) and positive emotions (*r* = 0.149, *p* < 0.05). The association between material wealth and negative emotions was not statistically significant. Similarly, socio-economic status significantly and positively correlated with mental health (*r* = 0.410, *p* < 0.01) and positive emotions (*r* = 0.152, *p* < 0.05), and negatively with negative emotions (*r* = −0.403, *p* < 0.01). Summarily, the mental health of retired individuals significantly associated with the quantity of material wealth and perception of their socio-economic status.

#### Physical health and sleep quality

As shown in **Table 6**, the coefficient of physical health (β = −2.832, SE = 1.283) implied that negative emotions decreased, on average, by 2.832 for each 1-unit increase in physical health while keeping other variables constant. The effects of sleep quality were also statistically significant on mental health (β = 0.483, SE = 0.228) and negative emotions (β = −4.592, SE = 1.161). This signified an increase in mental health by 0.483 and a decrease in negative emotions by 4.592 for each 1-point increase in sleep quality.

**In summary**, the above analyses show that gender, education level, physical health, sleep quality, material wealth, and socioeconomic status significantly affect the mental health of retired individuals. Among the control variables, female gender, physical health, sleep quality, and socioeconomic status can significantly reduce negative emotions, whereas education level, material wealth, and socioeconomic status could effectively enhance positive emotions. After adding other variables, the effects of sex, physical health, sleep quality, and socioeconomic status on mental health remained statistically significant.

### Effects of participation in CSOs on mental health

Participation in CSOs was defined in multiple dimensions: diversity, frequency, quality, and type. We conducted binary correlation analysis to show raw correlations between each dimension of participation and mental health, as shown in Table 4. We adopted regression models to estimate the net effect of each dimension, as shown in **Table 6**.

**Table 4:** Correlation among dimensions of participation and mental health.

	**Mental health**	**Positive emotions**	**Negative emotions**
Diversity of participation	0.145^*^	0.040	−0.141
Frequency of participation	0.274^*^	0.088	−0.235^*^
Quality of participation	0.317^*^	0.161*	−0.272^*^

* *p* < 0.1. The values in the cells are the Pearson's correlation coefficients among variables.

#### Diversity and frequency of participation

Diversity referred to the number of categories of CSOs who participated^2^. The frequency of participation referred to the number of times per week individuals participated in activities organized by CSOs. As shown in Table 4, diversity of participation positively and weakly correlated with mental health (*r* = 0.145, *p* < 0.1). The frequency of participation affected mental health positively and moderately (*r* = 0.274, *p* < 0.1), while negatively affecting negative emotions (*r* = −0.235, *p* < 0.1). As shown in Table 6, the coefficient of frequency (β = 0.481, SE = 0.215) implied that mental health increased on average by 0.481 for each 1-unit increase in the frequency of participation, while the net effect of diversity of participation on mental health was not statistically significant.

#### Quality of participation

Quality of participation was defined as the level of basic interpersonal needs, including belonging, dominance, and affection needs individuals acquired in CSOs. This determines the resilience and sustainability of organizations, according to interpersonal theory of behavior. As shown in Table 4, the quality of participation correlated: moderately and positively with mental health (*r* = 0.317, *p* < 0.1), weakly and positively with positive emotions (*r* = 0.161, *p* < 0.1), and moderately and negatively with negative emotions (*r* = −0.272, *p* < 0.1). Furthermore, as shown in Table 6, the net effects of participation quality remained statistically significant in the regression models, with coefficients of 0.158 (SE = 0.047), 0.907 (SE = 0.393) and −0.523 (SE = 0.24) in the mental health, positive emotions and negative emotions models, respectively. The results indicated that, on average, mental health and positive emotions increased by 0.158 and 0.907, respectively and decreased by 0.523 for each 1-unit increase in the quality of participation.

#### Types of participation

Most empirical studies tend to consider CSOs as a unique type of social interaction of retired adults; however, the types of CSOs are discussed to a lesser extent. There were four main types of CSOs in urban China: hobby CSOs such as plaza dancing groups (guangchangwu dui), health CSOs such as community care centers (laonianren zhaoliao zhongxin), study-related CSOs such as community seniors universities (laonian daxue), and semi-official CSOs referred to as the seniors associations (laonian xiehui).

We conducted T-Test analysis to compare the means of mental health between participants and non-participants, as shown in Table 5. Structural differences appear among several types of CSOs.

**Table 5:** Difference in mental health among participants and non-participants in CSOs.

**Mean-diff**	**Hobby-related CSOs**	**Health-related CSOs**	**Study-related CSOs**	**Semi-official CSOs**
Mental health	1.549^***^	0.958^**^	0.877^*^	−0.233
Positive emotions	5.105	7.301^**^	−0.443	−0.939
Negative emotions	−7.004^***^	−2.248	−4.893^**^	1.004

* *p* < 0.1,

** *p* < 0.05,

*** *p* < 0.01. The mean-diff values in cells indicate the comparison of means between those who participated in specific CSOs and those who did not.

Compared to non-participants, the mean difference in mental health for participants was: 1.549 (*p* < 0.01), 0.958 (*p* < 0.05) and 0.877 (*p* < 0.1) for hobby, health- and study-related CSOs. These results indicated that the average mental health of those participants of hobby-, health-, and study-related CSOs were 1.549, 0.958, and 0.877 points higher respectively, than non-participants. The mean difference of positive emotions was 7.301(*p* < 0.05) for participants of health CSOs. For negative emotions it was −7.004 (*p* < 0.01) and −4.893 (*p* < 0.05) for participants of hobby- and study-related CSOs.

In the multivariable models shown in Table 6, the effects of participation in the following types of organization were significant: health CSOs in the mental health model (β = 1.395, SE = 0.62), positive emotions model (β = 14.751, SE = 5.166), and study-related CSOs (β = 1.164, SE = 0.589) in the mental health model. Among the various types of CSOs, participation in health CSOs plays the most robust role in improving mental health and positive emotions.

**Table 6:** Results of multivariable regression analysis.

	**Model 1**	**Model 2**	**Model 3**
	**Mental health**	**Positive emotions**	**Negative emotions**
Gender	0.901^**^	0.928	−5.714^**^
	(0.527)	(4.393)	(2.683)
lnage	−1.353	−13.31	−0.988
	(2.279)	(18.999)	(11.602)
Edu_1	−0.004	−0.89	−2.053
	(0.707)	(5.894)	(3.599)
Edu_2	−0.342	−5.311	−1.288
	(0.502)	(4.183)	(2.554)
Physical health	0.113	−2.987	−2.832^**^
	(0.252)	(2.101)	(1.283)
Sleep quality	0.483^**^	−3.205	−4.592^***^
	(0.228)	(1.901)	(1.161)
Material health	0.125	1.583	−0.023
	(0.131)	(1.096)	(0.669)
SES	0.331^***^	1.019	−1.667^***^
	(0.122)	(1.017)	(0.621)
Diversity	−0.585^**^	−4.638	1.106
	(0.364)	(3.039)	(1.856)
Frequency	0.481^**^	1.008	−1.645
	(0.215)	(1.797)	(1.097)
Quality	0.158^***^	0.907^**^	−0.523^**^
	(0.047)	(0.393)	(0.24)
Hobby-related CSOs	0.33	7.365	1.488
	(0.674)	(5.615)	(3.429)
Health-related CSOs	1.395^**^	14.751^***^	−0.59
	(0.62)	(5.166)	(3.155)
Study-related CSOs	1.164^**^	5.075	−3.457
	(0.589)	(4.908)	(2.997)
Semi-official CSOs	−0.123	2.212	1.45
	(0.679)	(5.659)	(3.456)
_cons	15.749	102.217	81.959
	(9.835)	(81.99)	(50.068)
Observations	170	170	170
R^2^	0.342	0.169	0.361
Adjust R^2^	0.278	0.088	0.298
F	6.378^***^	2.514^**^	7.323^***^

* *p* < 0.1,

** *p* < 0.05,

*** *p* < 0.01.

The values in () are standardized errors of coefficients.

### The mediating mechanism of interpersonal needs on mental health

**Figure 2:**
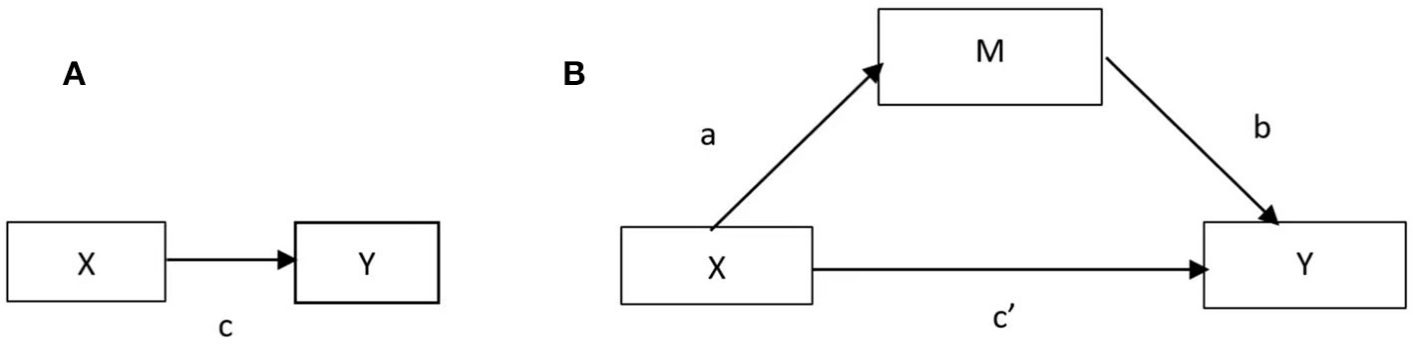
**(A)** Illustration of a direct effect. **(B)** Illustration of a mediation design.

The study further investigated the mediating mechanism of interpersonal needs on mental health acquired from participation in CSOs, testing whether diversity and frequency of participation affected mental health through the mediating role of interpersonal needs. As shown in Figure 2, we conducted two mediation analyses by conducting structural equation modeling to assess the mediating effect of interpersonal needs, using the Sobel test (62), which enhanced the robustness of the Baron and Kenny method (BK). The independent variables were diversity (X_1_) and frequency (X_2_) of participation, the mediating variable was interpersonal needs (M), the dependent variable was mental health (Y), and the control variables were the remaining variables in Model 1.

**Figure 3:**
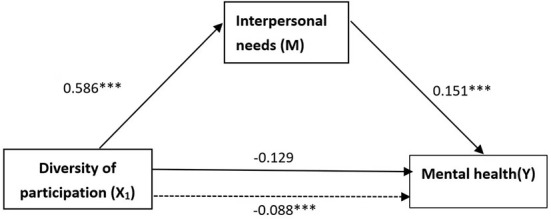
The mediation model associating diversity of participation with mental health. Dotted line denotes the original effect of the independent variable excluding the mediator variable. ^***^p < 0.01.

In the diversity model, as shown in Figure 3, the effect of X_1_ on M, the effect of M on Y, the effect of X_1_ on Y without controlling M, and the effect of X_1_ on Y controlling M were 0.586, 0.151, 0.88 and −0.129, respectively. The results of the Sobel test, Sobel's Z = 2.045, *p* < 0.05, and the results of bootstrap resampling procedures using 170 samples equal to the observations of the sample, unstandardized indirect effect = 0.088, SE < 0.052, Z = 1.825, *p* < 0.1, 95% bias-corrected bootstrap CI (0.012, 0.220) excluding a zero value, confirmed that “interpersonal needs” was a mediator between the diversity of participation and mental health of retired adults. Furthermore, the direct effect of X_1_ was significant at the 99% confidence level, and indirect effect became insignificant after adding the mediator variable. This result indicated a complete mediation mechanism of interpersonal needs associating diversity of participation with mental health.

**Figure 4:**
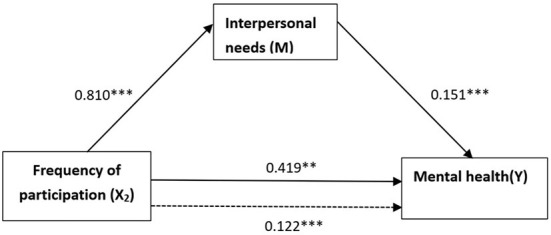
The mediation model associating frequency of participation with mental health. Dotted line denotes the original effect of the independent variable excluding the mediator variable. ^**^p < 0.05, ^***^p < 0.01.

In the frequency model, as shown in Figure 4, the effect of X_2_ on M, the effect of M on Y, the effect of X_2_ on Y without controlling M, and the effect of X_2_ on Y controlling M were 0.810, 0.151, 0.122 and 0.419 respectively. The results of the Sobel test, Sobel's Z = 2.097, *p* < 0.05, and the results of bootstrap resampling procedures using 170 samples, standardized indirect effect = 0.122, SE = 0.070, Z = 1.869, *p* < 0.1, 95% bias-corrected bootstrap CI (0.021, 0.299) excluding a zero value, confirmed that “interpersonal needs” also served as a mediator associating frequency of participation with mental health. Furthermore, the direct and indirect effects of X_2_ were both significant after adding the mediator variable, and the ratio of indirect effect to total effect was about 23%. The results showed a partial mediation mechanism of interpersonal needs associating frequency of the participation with mental health.

## Discussion

Although there is increasing number of CSOs in urban China with massive urbanization over the last 40 years, the value they generate for public mental health remains implicit. Previous studies have focused on its role in supplying public services and satisfying physical needs, as the Chinese government has been promoting the purchase of social service contracting policies since 2000, impelled by the new public management reform. However, CSOs also satisfy their psychological needs in an intangible way. This study examined the association between CSOs and mental health and investigated how membership and participation in various CSOs benefited retired adults' mental health.

### Effects of participation in CSOs on mental health

This study's first finding was that participation in CSOs benefited mental health by constituting social networks among retired adults in urban China. Some studies indicated that social interaction had a significant impact on retired adults' mental health through sharing social network resources (33, 63). Moreover, social networks could effectively alleviate the negative mental states of anxiety, depression, and interpersonal sensitivity and enable retired adults to maintain a positive mental status and enhance their subjective wellbeing (64). However, social networks among acquaintances such as family and friendship networks were emphasized more in Chinese research because the acquaintance society was a typical feature of Chinese traditional culture, where the networks among strangers were considered weak and incredible. This study confirmed that participation in CSOs could promote the formation of social networks based on variational residence in urban China, thus improving mental health, promoting positive emotions, and alleviating negative emotions in retired adults.

More detailedly, the frequency and quality of participation in CSOs could significantly improve mental health, by 0.481 and 0.158, respectively. The more individuals spent time in CSOs, the more they obtained a sense of belonging, dominance, and affection by participating in CSOs, they were likely to have more mental health. Effects of diversity of participation on mental health was absorbed by the quality of participation as the complete mediator effects were confirmed in the diversity SEM. Analysis of positive and negative emotions showed that the quality of participation in CSOs could significantly promote positive emotions and alleviate negative emotions among retired adults.

Effects of types of participation in various CSOs were also examined, and the findings differed from research in other countries. Health CSOs, particularly community care centers that supply physical therapy services and health care, and Study-related CSOs, mainly as community seniors universities exclusive for older adults, can satisfy psychological needs and promote mental health, while religion organizations were discussed and emphasized in other studies. Compared to non-participants, those who participated in health and study-related CSOs had higher scores on mental health by 1.395 and 1.164, respectively. Analysis still indicated that membership of health CSOs could exert significant effects on the positive and negative emotions of retired adults. It was worth pointing out that there was no significant difference between participants and non-participants of semi-official CSOs which were mainly organized and financed by the municipal government in mental health.

### Mediating effects of interpersonal needs

The mediating mechanism of interpersonal needs associated with participation in CSOs' mental health was significant. According to social capital theory, previous studies have focused on resources affiliated with social networks. This study illustrated the causal chain between participation in CSOs and mental health through the mediator of interpersonal needs, which made participation valuable for mental health among retired adults. The psychologist, W. Schutz proposed a three-dimensional interpersonal behavior theory based on interpersonal needs, highlighting that all individuals were eager for three basic needs in the process of interpersonal interaction: inclusion, dominance, and affection. These basic interpersonal needs determined the behaviors that individuals adopted in interpersonal interactions and how they described, interpreted, and predicted others' behaviors. CSOs provided basic needs in participation and interpersonal interactions, which helped to boost positive emotions, decrease negative emotions, and improve aggregating mental health.

## Conclusion

The present study was one of the first studies to explore the mechanisms of CSOs participation affecting the mental health of retired adults. Furthermore, it examined the mediating role of interpersonal needs in the association between CSOs participation and mental health, based on social capital theory. The major empirical findings discussed above verify these hypotheses and advance our knowledge of the association among CSOs, interpersonal needs, and mental health. First, CSOs participation had a significant positive effect on the mental health of retired adults. However, unlike religious organizations which play a strong role in other countries, the effect of health organizations was the strongest in China. Additionally, participation in a semi-official organization did not work, which was surprising. Second, the results highlighted that interpersonal needs mediate the association between CSOs participation and mental health among Chinese retired adults. When retired adults obtained the three dimensions of interpersonal needs: inclusion, dominance, and affection from participation, their mental health would be significantly enhanced. Additionally, the diversity and frequency of participation could facilitate the interpersonal needs of retired adults obtained from CSOs. In conclusion, this study showed that both CSOs participation and interpersonal needs were promoting factors in mental health. This indicated that the design of programs and policies for retired adults should consider meeting their interpersonal needs and involving retired adults in more CSOs participation that focus on promoting active aging. Of note, more high-quality longitudinal studies should be conducted to further substantiate the results of this study.

## Data availability statement

The original contributions presented in the study were included in the article/supplementary material, further inquiries can be directed to the corresponding author/s.

## Ethics statement

The studies involving human participants were reviewed and approved by Shenzhen University (PN-202200090). The patients/participants provided their written informed consent to participate in this study.

## Author contributions

YL made contribution to research design, theoretical analysis, and paper revision. JLa contributed to original draft preparation, data analysis, design of participation scale, and paper revision. JLi contributed to paper writing and revision. All authors contributed to the article and approved the submitted version.

## Footnotes

^1^The Chinese expression of community seniors universities is listed in (laoniandaxue), similarly hereinafter.

^2^For example, if a respondent participates in five organizations of the same category, it is considered one category, and the value of the diversity variable is recorded as one.
